# New Adsorption Materials for Deep Desulfurization of Fuel Oil

**DOI:** 10.3390/ma17081803

**Published:** 2024-04-14

**Authors:** Xiaoyu Qiu, Bingquan Wang, Rui Wang, Ivan V. Kozhevnikov

**Affiliations:** 1 School of Environmental Science and Engineering, Shandong University, No. 72 Seaside Road, Qingdao 266237, China; 2 School of Chemistry and Molecular Engineering, Qingdao University of Science and Technology, Qingdao 266042, China; 3 Department of Chemistry, The University of Liverpool, Liverpool L69 7ZD, UK; i.v.kozhevnikov@liverpool.ac.uk

**Keywords:** adsorption desulfurization, energy saving, adsorption materials, clean fuel

## Abstract

In recent years, due to the rapid growth of mankind’s demand for energy, harmful gases (SOx) produced by the combustion of sulfur-containing compounds in fuel oil have caused serious problems to the ecological environment and human health. Therefore, in order to solve this hidden danger from the source, countries around the world have created increasingly strict standards for the sulfur content in fuel. Adsorption desulfurization technology has attracted wide attention due to its advantages of energy saving and low operating cost. This paper reviewed the latest research progress on various porous adsorption materials. The future challenges and research directions of adsorption materials to meet the needs of clean fuels are proposed.

## 1. Introduction

**Figure 1:**
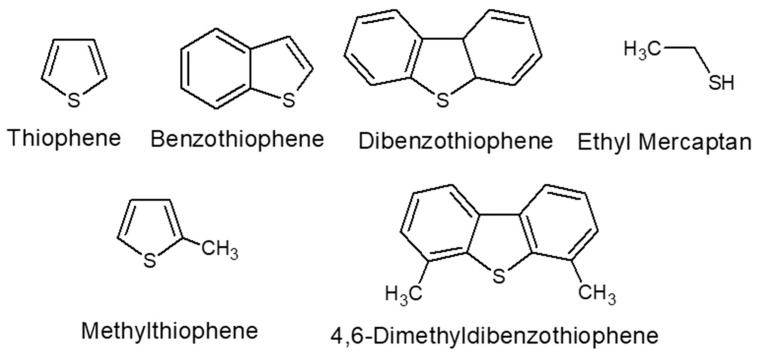
Common sulfur-containing compounds in fuels. Reprinted with permission from American Chemical Society [2].

Energy is the driving force behind the development of modern society. With the sweeping progress of industrialization, socio-economic development has been rapid, and the living standards of human beings have been greatly improved. At the same time, countries around the world have also expanded their demand for fossil energy [1]. Although the new energy industry has been booming in recent years, crude oil is still the main component of today’s energy, accounting for more than one-third of the world’s primary energy demand, according to the current technology and supply level. However, fuel oil has a high sulfur content, mainly including thiophene (TH), benzothiophene (BT), dibenzothiophene (DBT) [2], and their derivatives (see Figure 1). Sulfur, which threatens human health, affects the service life and performance of automobile engines, and damages refinery equipment, is considered to be one of the most dangerous pollutants in fuel oil. These deleterious sulfur compounds produce large amounts of sulfur oxides (SOx) during combustion [3]. The release of these harmful gases into the air can lead to environmental problems such as acid rain and haze, reducing air quality and soil fertility [4,5]. A survey by the World Health Organization (WHO) showed that long-term human exposure to high concentrations of SOx can lead to lung-related respiratory problems and pose a serious threat to the human respiratory system and nervous system. With the increasing demand for fuel and the gradual enhancement of national environmental awareness, various countries and regions around the world have implemented strict fuel sulfur limit standards. Relevant regulatory agencies have set the minimum allowable sulfur concentration in transportation fuels at 10 ppmw and 15 ppmw for gasoline and diesel, respectively [6,7]. Against this background, the development of low energy consumption and efficient ultra-clean fuel production technology has become one of the key research directions in the petrochemical industry.

## 2. Desulfurization Process

**Figure 2:**
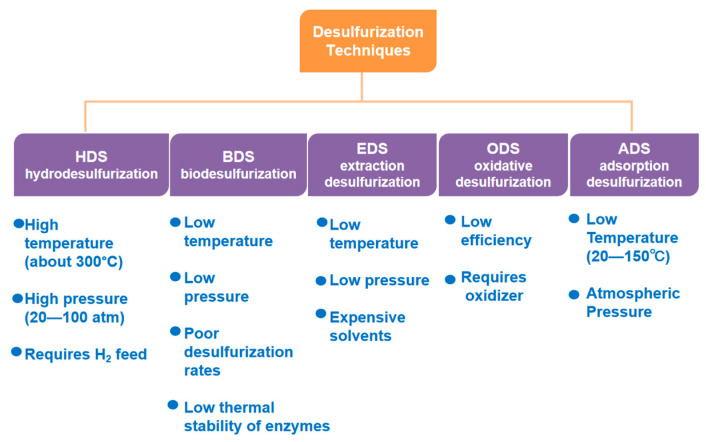
Overview of desulfurization technologies and their strengths and weaknesses.

Sulphur-containing compounds in fuel oils mainly include aliphatic organic sulfides such as mercaptans, sulfur ethers, disulfides, and in particular aromatic heterocyclic sulfur-containing compounds such as TH, BT, and DBT and their respective alkyl-substituted derivatives, which are difficult to remove. They must be further removed in accordance with industrial requirements and applicable regulations [8]. Therefore, the development of a sustainable and efficient desulphurization technology is still a hot area of scientific research. There are several ways to remove sulfur from liquid fuels, such as hydrodesulfurization (HDS) [9], biodesulfurization (BDS), extraction desulfurization (EDS), oxidative desulfurization (ODS) [10,11,12], and adsorption desulfurization (ADS) [13,14] (see Figure 2).

### 2.1. Hydrodesulfurization

**Figure 3:**
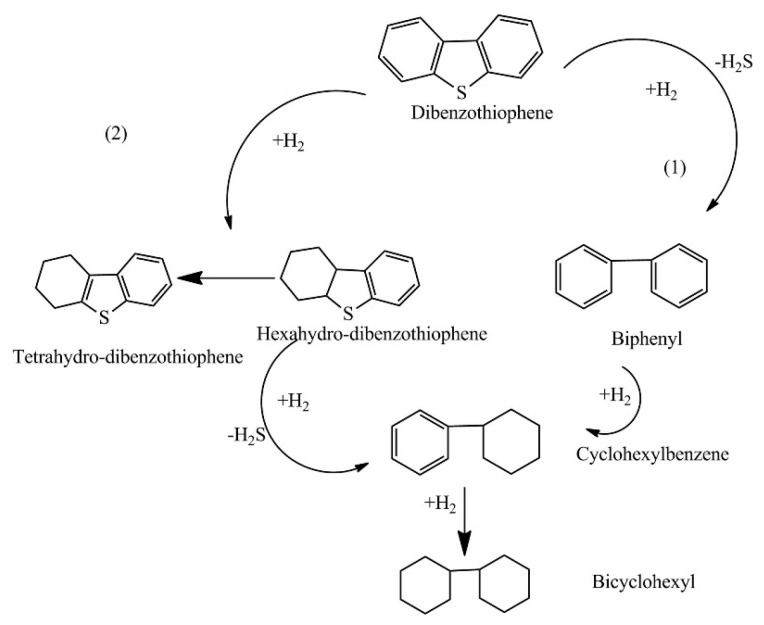
Possible pathways for the HDS of DBT. The first pathway (1) is direct desulfurization, where the sulfur atom is eliminated directly from the DBT molecule and biphenyl is formed. The second pathway (2) involves hydrogenation, where the DBT molecule is partially hydrogenated before the sulfur is eliminated by hydrogenolysis of the CS bond. Reprinted with permission from Elsevier [18].

HDS is the earliest and most mature desulfurization process [15]. As one of the important processing techniques in the current petroleum refining process, HDS has been able to meet previous limits on sulfur content in the production of clean fuels. HDS is a process that removes the sulfur impurities directly by converting the sulfur impurities to gaseous H_2_S through a hydrogenation reaction under the action of catalysts. It is worth noting that hydrogen from the catalytic reformer greatly facilitates hydrodesulfurization, enabling an efficient conversion of organic sulfur compounds to H_2_S and sulfur-free compounds to reduce sulfur content [16,17]. Taking the reaction of HDS with DBT as an example (see Figure 3), the first pathway (1) involves direct desulfurization, in which sulfur atoms are eliminated directly from the DBT molecule. The second pathway (2) involves hydrogenation, in which the DBT molecule is first partially hydrogenated and then the sulfur is eliminated by hydrogenolysis of the C-S bonds [18].

Although aliphatic organic sulfides such as mercaptans, sulfur brews, and disulfides can be efficiently removed from fuel oil by HDS technology, the removal of thiophene sulfur impurities is not satisfactory. Among the various types of sulfides contained in fuel oil, thiophene sulfides are the least active in the HDS process due to the effect of site resistance. The proportion of thiophene sulfides in the total sulfides in the fuel is more than 80%. Consequently, this feature of HDS makes the cost of using this technology to produce clean fuels with a sulfur content less than 10 ppm increase dramatically. At the same time, the olefins in the oil will be saturated with hydrocarbons, thus significantly reducing the octane number of the fuel, severely limiting its development and application in the present era.

### 2.2. Biodesulfurization

Biodesulfurization (BDS) refers to the removal of sulfur-containing compounds from fuels by converting them into water-soluble substances using microbial metabolic processes. Due to the high specificity of enzymes in microorganisms, BDS has a high selectivity that is unmatched by other desulfurization technologies [19,20]. In general, most microorganisms isolated from natural habitats do not specifically remove sulfur from fuel oil, but several bacterial species can achieve the utilization of sulfide as an energy source. For example, some isolated microorganisms utilize thiophene as a carbon and sulfur source [21].

BDS is known as a green method of desulfurization, but the weakness of catalyst specificity as well as the slowness of biological metabolism still exist in desulfurization. Although the efficiency of enzymatic desulfurization has been improved in the literature through the modification of and support from different forms of enzymes, its desulfurization performance is almost entirely dependent on the activity of the enzyme, and the dynamic reaction tolerance of the enzyme. This is highly operationally difficult for industrial production and its precision also requires further experimental research and exploration.

### 2.3. Extraction Desulfurization

In general, the polarity of sulfur-containing compounds is stronger than that of other hydrocarbons in the fuel. For this reason, the appropriate polar solvent can be selected according to the principle of “similar solubility” [22]. By mixing it with the fuel oil and transferring the sulfur compounds from the fuel oil to the polar solvent with the help of the strong solubility of sulfur compounds in polar solvents, and then separating the polar solvent containing sulfur compounds from the fuel oil, the removal of sulfur compounds from the fuel oil is realized. This method is called extraction desulfurization technology [23,24].

Clearly, the excellent performance of the polar extractant is the key to the whole technology. The most widely studied extractants used in extractive desulfurization include organic solvents and ionic liquids (ILs), but it is necessary to extract the fuel several times to achieve the effect of deep desulphurization, and it may affect the quality of the fuel [25]. Compared with other desulfurization technologies, EDS does not react with hydrocarbons, and does not affect the chemical structure of the compounds in fuel oil [26]. However, some aromatic hydrocarbons in fuel oil have a similar polarity to organic sulfides, which leads to a large loss of fuel oil volume during the extraction process. Secondly, some organic solutions in the extraction solution will have a certain corrosive effect on the extraction device and are difficult to recover in the regeneration process. Finally, the extraction process for ionic liquids often needs a high temperature to assist it, and it takes a long time to reach equilibrium. Moreover, the cost of ionic liquid is high, so its thermal stability and chemical stability still need to be further improved to reduce operating costs [27].

### 2.4. Oxidative Desulfurization

The C-S bond within organic sulfide in fuel oil is almost non-polar, and its solubility in water or other polar solvents is similar to those of its corresponding hydrocarbons [28]. Therefore, the oxygen atom can be attached to the sulfur atom of the organic sulfide by means of selective oxidation to increase its dipole moment and make it soluble in organic solvents [29,30,31]. Therefore, they can be readily removed from the fuel oil by adsorption, extraction, and distillation, etc., to achieve deep desulfurization. The ODS reaction can be performed at room temperature and pressure without the need for an expensive hydrogen source [32,33].

Although ODS has many advantages, there are still some difficulties in industrial application [34]. For example, it consumes a lot of energy and needs a large acoustic wave device, which increase the investment cost [35,36]. Moreover, the selectivity and regeneration capacity of the catalyst and the storage and transportation of the oxidant, as well as the influence of the oxidative desulfurization process on the quality of fuel oil, need to be further explored in practical industrial applications [37,38,39].

### 2.5. Adsorption Desulfurization

In contrast, adsorption desulfurization technology seems to bring to light the deep desulfurization of fuel oil. Currently, ADS is a low-cost alternative to HDS, in which a suitable adsorbent is added to the fuel, and the sulfide in the fuel is immobilized on the surface or inside of the adsorbent through certain chemical or physical effects, thus removing the sulfide from the fuel. ADS has the advantages of mild reaction conditions, low operating costs, almost no change in the composition of oil, and a minimal loss of gasoline octane rating [40]. In addition, according to the requirements of adsorbents for important characteristics such as a high surface area, large pore volume, good structural strength, stability, and more mesopore, ADS is widely used to selectively remove sulfides from liquid fuels [41,42]. More importantly, sulfur-containing adsorbents are regenerated for reuse by the elution of sulfur-containing substances under other conditions. According to the different forces between adsorbents and sulfides, fuel oil adsorption desulfurization technology can usually be divided into reactive (chemical) adsorption desulfurization and non-reactive (physical) adsorption desulfurization.

In the process of desulfurization by physical adsorption, the sulfur-containing components are adsorbed by van der Waals forces between the sulfur-containing compounds and the functional groups on the surface of the adsorbent [7]. Naturally, this kind of adsorption is closely related to the pore size structure and specific surface area of the adsorbent. For instance, Jeon et al. [43] prepared carbon-based adsorbents with tunable porosity and determined the adsorption desulfurization capacity of adsorbents with different pore structures. It was found that, when the pore size was concentrated at 0.6–1.2 nm, it had the strongest adsorption capacity for organic sulfides. This indicates that suitable pore size conditions can promote a better physical adsorption performance. Although physical adsorption is fast, it cannot selectively adsorb aromatic sulfur compounds due to the generation of van der Waals forces, resulting in poor selectivity and an unstable adsorption of aromatic sulfur compounds that cannot be formed industrially.

**Figure 4:**
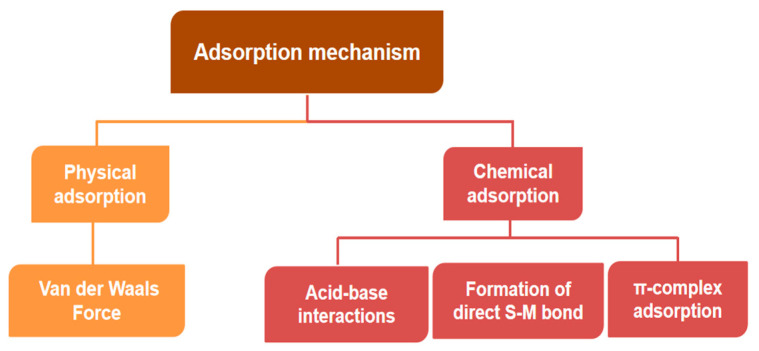
Diagram of adsorption mechanism.

Chemical adsorption is achieved through the complexation reaction between the adsorbent and the sulfur-containing compounds, resulting in chemical bonding (π bond/σ bond), thus realizing the adsorption of sulfur-containing components [18]. The specific desulfurization mechanisms of chemical adsorption are mainly acid–base interactions, formation of direct sulfur(S)–metal(M) bonds (σ), and π-complex adsorption (Figure 4). In the acid–base interaction mechanism, most thiophene sulfides in the fuel are Lewis bases, which are easily adsorbed by Lewis acid-type active sites. Therefore, materials that can form Lewis acid sites are generally selected as adsorbents to adsorb thiophene sulfides containing lone pair electrons in fuel. For instance, Xiong et al. [44] prepared boron nitride nanosheets with different degrees of boron defects using barbituric acid as a co-reactive source. By changing the electronic structure of BN, the conduction band of BN decreased from −1.35 eV to −0.54 eV. Meanwhile, the hardness of the chemical acid was reduced, which made it easier for a soft-acid–soft-base interaction with DBT to occur. In order to enhance competitive adsorption with aromatic hydrocarbons, some reports have suggested that the adsorption selectivity can be improved through the formation of direct sulfur–metal bonds. This action is the direct formation of sulfur–metal σ-bonds by giving the lone pair of electrons from the sulfur atom in the organic sulfide to the metal atom of the adsorbent. Unsaturated metal sites have a strong affinity for sulfides and the direct formation of S-M bonds could enhance the desulfurization performance of the adsorbent. The π-complexation is realized by the formation of pi (π) complexes by electron transfer between the adsorbent and the reactants. Khan et al. [45] explored the effect of initial BT concentration on the adsorption capacity of MIL-47(V) and MIL-53(Cr). It was shown that acid–base interactions and n-complexation were the main forces involved in the process. Compared with physical adsorption desulfurization, this method is more selective for aromatic sulfur compounds. However, chemical adsorption desulfurization requires high reaction temperatures and the regeneration of the adsorbent is difficult, which needs to be calcined or washed with a specific solvent [1,46,47].

In brief, ADS can selectively adsorb aromatic sulfides, which are difficult to be removed by HDS, and reduce the sulfur content in fuel without sacrificing the oil. More importantly, this technology is able to achieve efficient removal of recalcitrant sulfides under mild conditions. ADS stands out from many other desulfurization methods due to its easy operation, low operating cost, green environment, and mild reaction conditions, and has received extensive attention from researchers and resulted in many studies. ADS has been recognized as one of the most cost-effective technologies for reducing the sulfur content of fuel. At present, the industrial adaptability of adsorption desulfurization is extremely limited, and it is necessary to find a green adsorbent with a lower cost and higher efficiency.

## 3. Different Types of Adsorption Materials

In the adsorption desulfurization system, the selection of the adsorbent is crucial, which is related to the desulfurization efficiency of the whole system. The efficiency of adsorption desulfurization is mainly determined by the adsorption capacity, selectivity, regeneration performance, and stability of the adsorbent. At present, the main absorption materials used in the field of adsorption desulfurization are activated carbon materials, zeolite molecular sieves, metal–organic framework materials, transition metal oxides, and so on.

### 3.1. Activated Carbon Materials

Active carbon materials have the characteristics of a large specific surface area, stable structure, and low preparation cost. They are a kind of adsorbent commonly used in industry, and have a high adsorption capacity and good application potential for the desulfurization of high-sulfur-content oil products. There are two main adsorption desulfurization mechanisms of carbon materials. One is to use the surface chemical properties and pore structure of porous carbon itself, or to modify the surface properties of porous carbon through activator activation, doping heteroatoms, etc., to improve the adsorption desulfurization performance. During the adsorption process, the plane of organic sulfur can produce π-π stacking adsorption with the conjugated aromatic ring π plane on the surface of the active carbon material. At the same time, the surface pore structure and surface heteroatomic functional groups of carbon also affect the adsorption of organic sulfur [14,48]. It is worth noting that, for unmodified activated carbon, the adsorption desulfurization effect is generally poor.

As such, it is necessary to modify activated carbon to improve its adsorption performance. Wang et al. [49] used activated carbon as an adsorbent to investigate its adsorption and desulfurization performance on TP simulated oil (300 μg/g). The results showed that the sulfur adsorption capacity of activated carbon for TP was only 0.04 mmol (S)/g adsorbent. In order to improve the adsorption capacity of activated carbon, it is generally necessary to use oxidants, metal salts, and metal oxides to modify the activated carbon. For example, Xiong et al. [50] successfully prepared cerium-loaded activated carbon using the traditional impregnation method, and evaluated its effect on removing DBT (500 ppm initial sulfur concentration). The maximum adsorption capacity of C-BN-0.005 for DBT was 49.75 mg S/g adsorbent. The study confirmed that the addition of cerium to activated carbon can enhance the antioxidant capacity of activated carbon. At the same time, the adsorption selectivity and sulfur capacity of C-BN are higher than that of activated carbon, which is mainly due to the Lewis acid–base interactions. The regeneration capacity of C-BN was also evaluated, and the adsorption showed a small decrease. Therefore, the regenerated adsorbent still showed a good adsorption capacity.

**Figure 5:**
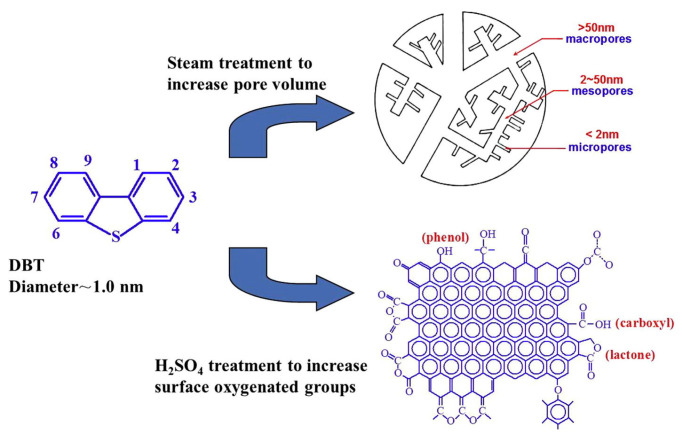
Modification of the pore structure and surface chemistry of activated carbon adsorbents. Reprinted with permission from Elsevier [51].

**Figure 6:**
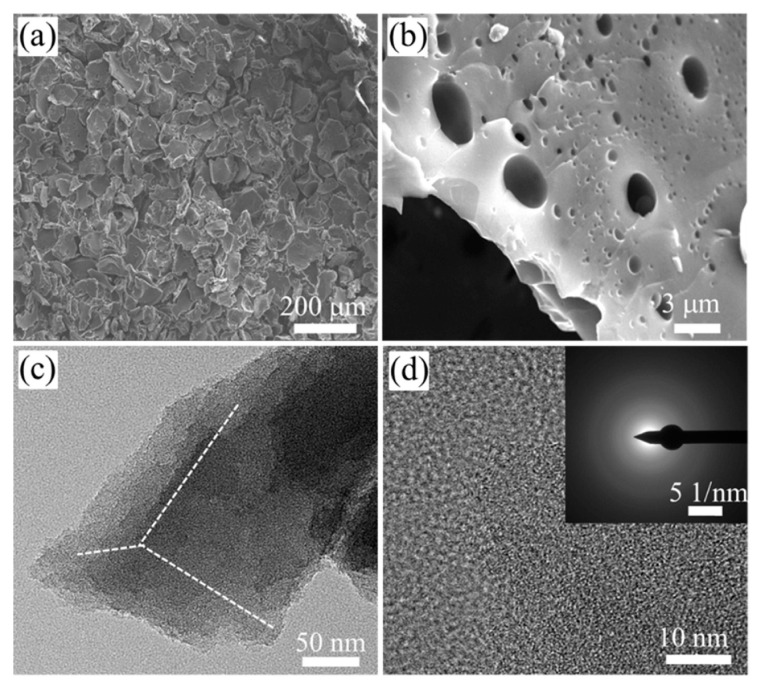
The TEM images (**a**,**b**) and HRTEM images(**c**,**d**) of DHPC-10 with graphite lattice stripes and a large number of pores. Reprinted with permission from Elsevier [52].

**Figure 7:**
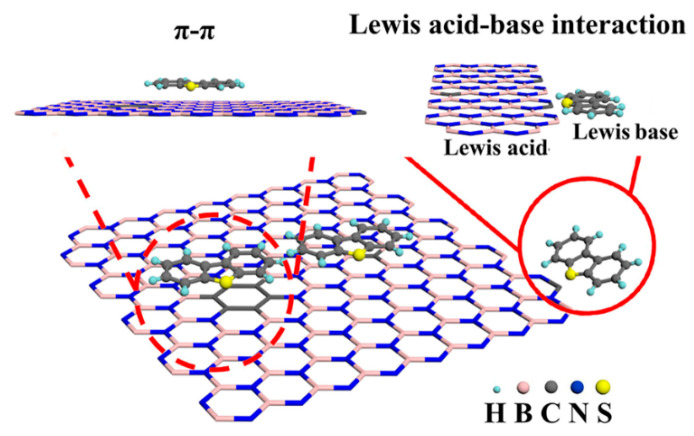
Possible adsorption mechanism of BCN aerogels. Reprinted with permission from Wiley [53].

It is worth noting that the pore structure of activated carbon itself is also an important factor to consider when investigating its role as an adsorbent. The adsorption capacity of a material is mainly affected by the volume of micropores. The micropores make the dispersive interaction dominant, while the mesoporous pores are mainly conducive to diffusion. In addition, specific interactions during the adsorption process also favor the adsorption of DBT and 4,6-DMDBT when the acidic groups are located in larger pores. Deng et al. [51] chemically treated activated carbon with water vapor and concentrated sulfuric acid to improve its pore size structure, and thus its efficacy as an adsorbent. As shown in Figure 5, the number of mesoporous pores after treatment with 900 °C steam increased from 0.248 to 0.856 cm^3^/g compared to untreated activated carbon. The increased pore volume can accommodate more surface oxygen-containing functional groups, increasing the final sulfur capacity from 10.9 mg/g to 20.6 mg/g. In general, the similar adsorption pattern of aromatic and DBT molecules on activated carbon induces a strong competitive adsorption between aromatic hydrocarbons and aromatic sulfur compounds. In the present study, due to the significant increase in surface oxygen-containing groups due to sulfuric acid treatment, direct interactions between adsorbed DBT molecules and the adsorbent’s surface may be formed through S atoms, and this particular adsorption pattern leads to a highly selective adsorption of DBT. Similarly, Wu et al. [52] synthesized a novel defect-rich hierarchical porous carbon (DHPCs) material from coal tar pitch (CTP) by oxidative pretreatment, melamine graft modification, and one-step carbonization activation. By adjusting the content of C-N bonds, the pyrolytic activation process of CTP was enhanced, which in turn affected the formation of grain defects and pores in DHPCs. In Figure 6, intersecting graphite lattice stripes and a large number of pores can clearly be observed, which can provide abundant adsorption sites. The circular selection region electron diffraction (SAED) diagram (Figure 6d) also reveals the presence of edges and defects. The adsorption data were obtained by adding different amounts of DHPCs to 5 mL of model oil with a 500 μg/mL initial concentration of sulfur. The maximum adsorption of DBT by DHPC-10 was shown to be up to 86.94 mg S/g and was maintained at 75.11 mg S/g after five cycles. Considering the possible strong competitive adsorption between aromatic hydrocarbons and carbon, DHPC-10 was used as an adsorbent for the adsorption and removal of DBT in a volume ratio of 9:1 of octane to p-xylene. Due to the competitive adsorption between DBT and p-xylene, the maximum adsorption capacity of DBT was only reduced to 73.82 mg S/g. This is because the micropores (V < 0.7 nm) with a similar dynamic diameter to the DBT molecules provide additional active sites for DBT adsorption, which becomes a critical factor for the high selectivity adsorption of DBT. Compared with intact graphitic carbon, the electron density of porous carbon with defects was significantly reduced. The resulting C atoms, which are partially positively charged, bind more easily to sulfide molecules. Moreover, due to the high electronegativity of nitrogen atoms, the π-π interaction between nitrogen-based polyporous carbon and DBT is enhanced, and the adsorption of DBT is promoted. In recent years, Luo et al. [53] prepared a series of new aerogel-like adsorption materials (BCNs) with three-dimensional interconnected porous networks by using glucose as carbon source and using hydrogel template and freeze-casting technology. The adsorption and desulphurization capacity of the prepared aerogel reached an unprecedented high level (30.8 mg S/g). It was shown that the specific surface area and Lewis acidic sites of BCN aerogels could be affected by adjusting the carbon content in the aerogels, which in turn affects the mass transfer rate and the Lewis acid–base interactions between DBT and BCN aerogels (see Figure 7). BCN-2.5 can also achieve highly selective adsorption of DBT in the presence of impurities such as aromatic hydrocarbons and nitrogen compounds. The concentration of distractor is 10% of the mass of sulfur. It is further proved that the adsorbent has good selectivity. Finally, the regeneration capacity of the material was evaluated, and the adsorption capacity of BCN-2.5 after regeneration was still 23.6 mg S/g.

In order to improve the selectivity of activated carbon materials, molecular imprinting technology has been applied to adsorb organic sulfur in fuel oil or a model solution. For example, Liu et al. [54] synthesized a molecularly imprinted polymer adsorbent using porous magnetic carbon nanospheres as the carrier and DBT as the template. The saturated adsorption capacity of the adsorbent for DBT (3.0 mmol/L) reached 32.00 mg S/g within 40 min, and it had a good selectivity for DBT. MIP/p-Fe_3_O_4_@C also has a strong regenerative capacity and stability. After five adsorption/desorption cycles, DBT’s rebinding capacity remains above 80%. Table 1 summarizes some recent examples of activated carbon materials used for the adsorption desulfurization of fuel oil.

Although activated carbon adsorbent materials are low cost and readily available, the large pore size distribution leads to limited mass transfer rates and reduced selectivity for desulfurization. Therefore, their performance can be improved by combining them with other types of adsorbents such as metal–organic frameworks or metal oxide nanoparticles. In addition, activated carbon materials are prone to structural damage during cyclic regeneration and are therefore less recyclable. Preparation parameters for carbon materials at a large batch scale also need to be optimized.

### 3.2. Zeolite Molecular Sieve Adsorbent

Molecular sieves, as a member of the crystallized silica-aluminate family, are formed from aluminum-oxygen tetrahedra or silica-oxygen tetrahedra by oxygen bridge bonding. Owing to their high specific surface area, well-organized pore structure, and excellent thermal stability, molecular sieves have become the most researched and widely used adsorbent materials [61,62]. In the application of liquid fuel adsorption and desulfurization, the interaction between molecular sieves and sulfides includes physical adsorption and chemical adsorption. According to the molecular characteristics of sulfide in oil, some researchers attributed their existing low sulfur adsorption capacity to the influence of adsorbent pore structure, which limited the mass transfer and adsorption properties of thiophene sulfide molecules. Therefore, they began to use mesoporous molecular sieves as the carrier to prepare adsorbent, and a lot of research has been carried out on this topic [63,64,65].

**Figure 8:**
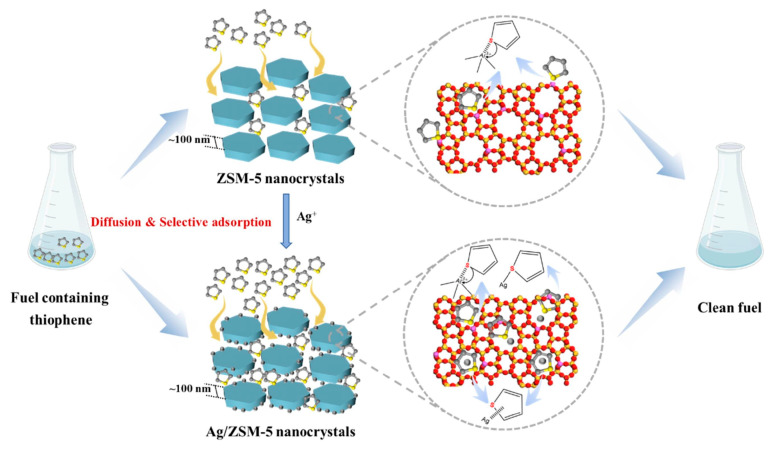
Adsorption mechanism of Ag/ZSM-5 nanocrystals. Reprinted with permission from Elsevier [68].

At present, the most common molecular sieves include FAU (NaY and NaX), ZSM-5, OMS-2, MMW, HMS, and so on [66,67]. However, since molecular sieves have almost no active sites, active groups or metal active centers need to be introduced to improve their catalytic activity during the adsorption and desulfurization process [68]. In recent years, metal-modified molecular sieves dominated by chemisorption have been widely reported for their excellent selectivity and high adsorbed sulfur capacity. Researchers generally load the transition metal active site into the structure of the molecular sieve using impregnation or the ion exchange method to improve its adsorption performance and adsorption selectivity. For example, Ahmadi et al. [69] successfully obtained a CoO-MSU-S adsorbent using a cobalt-modified mesoporous material, MSU-S. The results show that the impregnation of Co^2+^ can enhance the adsorption capacity of MSU-S. The pore structure of traditional ZSM-5 molecular sieves is narrow, which seriously affects the adsorption performance of thiophene sulfide in fuel. Xiao et al. [68] synthesized ZSM-5 zeolite nanocrystals using a steam-assisted gel crystallization strategy, and controlled the size of zeolite particles within 70 ~ 160 nm. Subsequently, a Ag/ZSM-5 adsorbent was successfully prepared by ion exchange of ZSM-5 nanocrystals with NH4^+^ and Ag^+^. The results show that the mesoporous properties and acid sites of the molecular sieve are enhanced, and the adsorption desulfurization performance is improved (see Figure 8). In order to investigate the regeneration performance of ZSM-5, dynamic adsorption penetration curves of 500 ppmw thiophene on ZSM-5 regenerated in a fixed bed were obtained. The saturated sulfur capacity of the nanocrystals was found to decrease slightly after several regenerations, which may be related to the ethanol treatment time and calcination temperature. Similarly, Wang et al. [70] synthesized a graded cheese-like C-ZSM-5 adsorbent material by acid-base treatment and introduced Ti as an active center into C-ZSM-5. It was found that the catalytic capacity of the new Ti/C-ZSM-5-30 (corrosion for 30 min) was 4.4 times higher than that of the conventional Ti/ZSM-5, and DBT (1000 ppmw) was completely removed after 60 min at 338 K. After acid–base modification, the Ti/C-ZSM-5 catalyst has more mesopores and a higher specific surface area, which increases the contact opportunities between active sites and reactants and improves the desulfurization rate of conventional ZSM-5. However, it difficult to make the active components uniformly distributed using this route, and it is easy to cause the phenomenon of molecular sieve pore blockage, which hinders the diffusion and mass transfer of the adsorbent. In addition, some researchers have modified zeolite molecular sieves with bimetal to improve their adsorption performance. For example, Dashtpeyma et al. [71] successfully prepared meso-CuCe and meso-NiCe adsorbents by modifying NaY-type zeolite from a microporous to a mesoporous structure using sequential acid–base treatments, and then impregnating it with bimetallic Cu-Ce and Ni-Ce. This study improved the pore structure of NaY and increased the number of active sites of metal cations. In the exchange and impregnation of many metal cations (Fe^3+^, Ag^+^, Cu^2+^, Cu^+^, Ce^4+^, Co^2+^, etc.), Cu^2+^ is widely used because of its good performance in the adsorption of thiophene sulfide by π complexation. Furthermore, the effect of adsorbent on the adsorption capacity of dibenzothiophene was investigated with the addition of toluene. When the concentration of toluene in the model fuel was 1000 ppm, the adsorption capacity decreased to 22.2 mg S/g. This is caused by the competitive adsorption between dibenzothiophene and toluene, as both have similar aromatic backbone structures. In this case, toluene competes with sulfur compounds and reduces their adsorption capacity. Table 2 summarizes some recent examples of molecular sieve materials used for the adsorption desulfurization of fuel oil.

Molecular sieves have the virtues of adjustable pore size, a large specific surface area, and good stability and reproducibility, making them one of the most advantageous adsorbent materials at present. Their desulfurization performance is closely related to the microstructure and surface properties of the molecular sieve carrier itself. Therefore, in the design and preparation process of molecular sieves, based on the properties of target sulfides, the crystallization process of molecular sieves should be controlled to make them have a rich pore structure. At the same time, the active components, acidity, and other surface properties of the molecular sieves should be precisely regulated, so that they can have both large sulfur capacity and high desulfurization precision and selectivity. This will be an important direction for future research. We predict that more and more functional molecular sieves will be applied to the study of fuel desulfurization in the near future.

### 3.3. Metal-Organic Frameworks

MOFs are crystalline porous materials composed of inorganic metal centers interlinked with organic ligands by self-assembly [78]. They have a periodic network structure different from that of ordinary inorganic porous materials and organic complexes. In the 1990s, Yaghi et al. [79] successfully synthesized nano-porous materials with organic ligands and inorganic metal ions or clusters forming intramolecular pores through coordination bond self-assembly and named them metal–organic frameworks (MOFs); MOFs have rapidly become a research hotspot [80,81]. They show great potential and have attractive development prospects in modern material research [82,83]. MOFs have been widely studied in the adsorption field because of their large specific surface area, suitable microporous pores, and highly ordered crystal structure, which can provide uniform and effective active sites [84]. By varying the types and proportions of organic ligands and metal ions, researchers have synthesized thousands of MOFs with different forms and functions. More notably, MOFs with very high specific surface areas have been synthesized in recent years, much higher than activated carbon and molecular sieve materials [85,86,87,88]. Nevertheless, single MOFs crystals are not stable enough and their adsorption properties need to be improved. As a result, researchers often employ various strategies to modify MOFs materials to enhance the performance.

**Figure 9:**
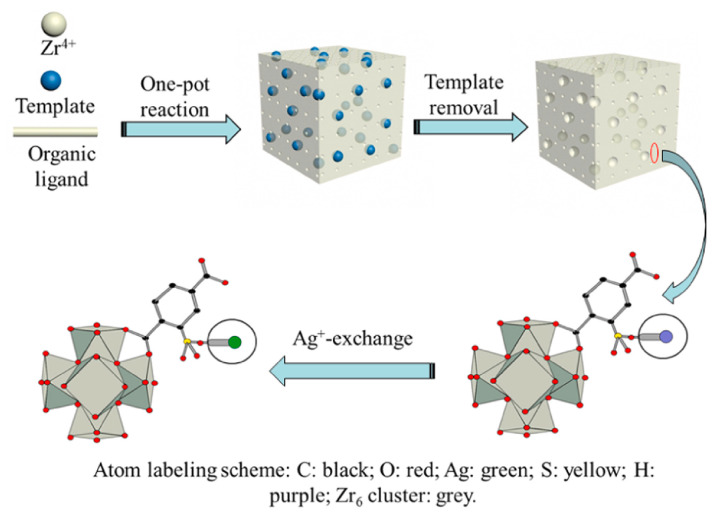
Schematic diagram of HP-UiO-66-SO_3_H preparation and Ag^+^-exchange. Reprinted with permission from Elsevier [90].

MOF-5 is considered to be a promising adsorption desulfurizer due to its large specific surface area. However, the stability of conventional MOF-5 is poor, which limits its desulfurization efficiency. Mguni et al. [89] synthesized a series of new (xNi/Zn)-BDC adsorption materials with different Ni contents using formic acid as a modulator, and studied the properties of these materials in model diesel ADS. The results showed that the addition of formic acid significantly increased the grain size and crystallinity of (xNi/Zn)-BDC materials. The adsorption experiment results showed that the overall adsorption activity of Ni-BDC increased by nearly two times after the synthesis of Ni-BDC with formic acid. The total adsorption capacity and partition coefficient of (xNi/Zn)-BDC adsorbents can reach 4.14 mg/g and 0.053 mg/g/ppm, respectively. Zhang et al. [90] developed a novel hierarchical pore UiO-66 based on MOF-5. Subsequently, Ag^+^ was introduced into UiO-66 using the ion exchange method to improve desulfurization (see Figure 9). The results show that HPUiO-66-SO_3_Ag has a higher adsorption energy and cycling properties for TH and BT. At an initial sulfur content of 200–1000 ppmw, the adsorption capacity of HP-UiO-66-SO_3_Ag-0.25 is about 31.4 mg S/g. In addition, HP-UiO-66-SO_3_Ag samples showed a high level of adsorption selectivity, and the adsorption capacity decreased by only 18.4% in the presence of aromatic competitors. The regeneration results showed that the initial adsorption capacity of HP-UiO-66-SO_3_Ag can be regenerated by about 90% after four cycles.

**Figure 10:**
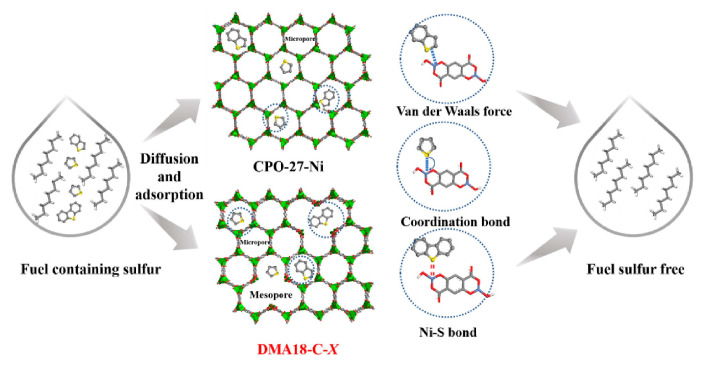
Schematic diagram of desulfurization mechanism of CPO-27-Ni and DMA18-C-X. Reprinted with permission from Elsevier [91].

In recent years, defective MOFs have been gradually applied in the adsorption desulfurization field because of their adjustability. In order to increase the number of active sites for adsorption desulfurization, Lu et al. [91] synthesized the defective CPO-27-Ni (DMA18-C-X) adsorption material using DMA18 as a template. The adsorption experiments showed that, when the molar ratio of DMA18 to Ni^2+^ was 0.5, the dynamic adsorption capacity of DMA18-C-0.5 for DBT, BT, and TP (100 ppmw) was 3.79, 6.45, and 9.29 times that of traditional CPO-27-Ni. The reasons for the enhanced adsorption desulfurization performance are shown in Figure 10. The mesoporous formation of DMA18-C-X material greatly enhances the mass transfer of sulfide in the adsorbent, weakens the effect of steric hindrance, effectively promotes the exposure of more unsaturated Ni^2+^ active sites, and enhances the full contact between Ni^2+^ active sites and sulfide. Finally, the sample showed an excellent regeneration performance. After three cycles of regeneration, the adsorption capacity of DMA18-C-0.5 was 86.30%, 85.59%, and 83.9% of the fresh samples’, respectively.

**Figure 11:**
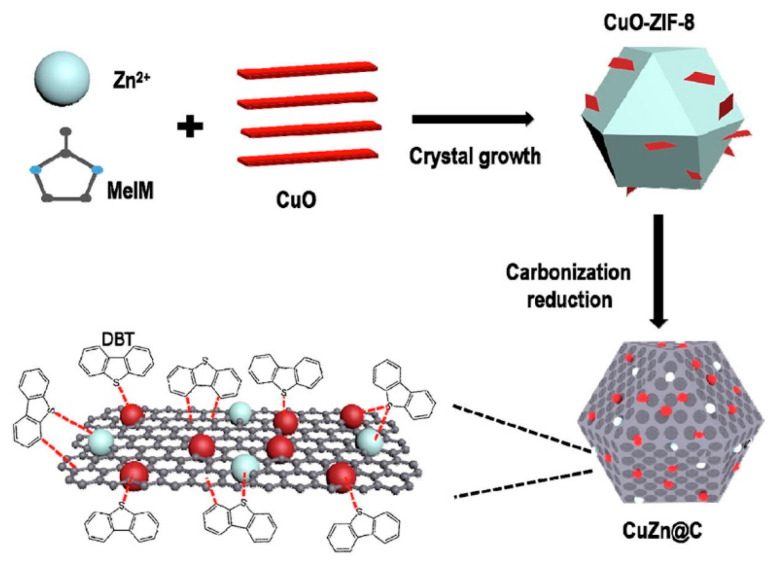
Schematic representation of preparation process of adsorbent Cu/Zn@C. Reprinted with permission from Elsevier [92].

In addition, MOFs can form composites with excellent adsorption properties in conjunction with other materials such as metals and nonmetallic compounds. For example, Huo et al. [92] first pre-grew ZIF-8 on CuO nanosheets, and then successfully synthesized Cu and Zn bimetallic doped porous carbon (CuZn@C) using the carbonization method. The preparation method is shown in Figure 11. The specific surface area of the adsorbent was increased via the evaporation of Zn in the process of high temperature carbonization. The Cu (I) obtained by Cu (II) reduction is uniformly dispersed and embedded in the carbon matrix derived from ZIF-8, effectively forming π-complexation with thiophene sulfur compounds. Due to the uniform distribution of bimetallic active sites and its excellent structure, the obtained CuZn@C has superior ADS properties. Chen et al. [93] successfully prepared a novel composite material, Fe_3_O_4_-ATP/MOF-199 (FAM), by compositing MOF-199, attapulgite (ATP), and Fe_3_O_4_ using the solvent-thermal method and used it for the removal of TP from model oils. MOF-199, as a porous material with a large specific surface area and a high porosity, can be accessed by TP to remove sulfur compounds using intermolecular forces. The addition of ATP changes the crystal structure of magnetic MOF-199, which brings sulfide closer to the active center of the adsorbent and improves its adsorption capacity and selectivity. However, due to the strong interaction between the metal center of the adsorbent and the sulfur atoms in thiophene, the active site of the adsorbent was reduced, and the repeated adsorption effect was poor. 

**Figure 12:**
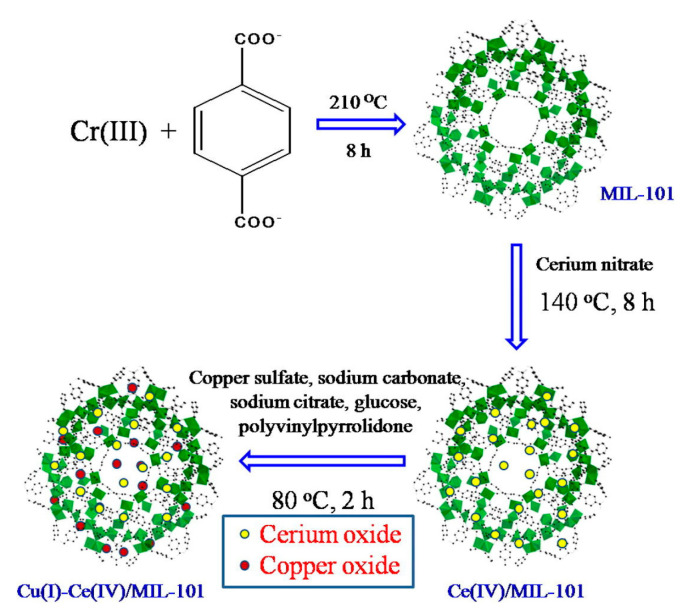
Illustration of the synthesis of Cu-Ce/MIL−101. Reprinted with permission from Elsevier [95].

With the increasing understanding of MOF materials, researchers have found that the addition of rare metals can also improve the adsorption properties of MOF materials [94]. Khan et al. [95] successfully loaded Cu_2_O and CeO into a highly porous MOF (see Figure 12). After the introduction of two metal oxides, MIL-101 retained its intact crystal structure. The adsorption capacity of Cu-Ce/MIL-101 for BT was 2.9 times higher than that of the original MIL-101 due to the synergistic effect of π-complexation between Cu(I) and BT and the direct bonding between Ce(IV) and S during BT adsorption. Notably, Cu-Ce/MIL-101 selectively adsorbed BT even in the presence of an aromatic additive (toluene). In addition, since Ce species can stabilize the oxidation state of Cu^+^, Cu-Ce/MIL-101 has excellent reusability. A summary of some MOFs adsorption materials performances in ADS is shown in Table 3.

In conclusion, MOFs have bright prospects as adsorption materials in the field of fuel oil desulfurization. However, there are still challenges such as high production costs, complex synthesis processes, structural vulnerability at high temperatures, and poor stability. Additionally, the presence of water molecules in the fuel oil creates a bottleneck against MOFs materials effectively utilizing metal active sites for sulfur adsorption. Even trace amounts of water molecules can compete with organic ligands and metal ions for adsorption coordination, thereby affecting the adsorption performance of MOFs materials towards thiophene-like sulfides. Therefore, optimizing synthesis procedures, enhancing sulfur adsorption capacity, and improving desulfurization selectivity and stability are crucial issues that need to be addressed for future development of MOFs materials.

### 3.4. Metal Oxide-Based Adsorbent

**Figure 13:**
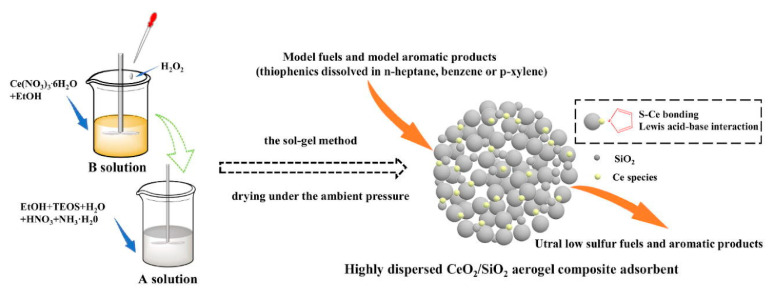
Preparation process and action mechanism of CeO_2_/SiO_2_ aerogel. Reprinted with permission from Elsevier [110].

Metal oxides (Fe_3_O_4_, MnO, CuO and ZnO, etc.) are early adsorbent materials used for fuel desulfurization [40,102,103,104]. They mainly produce metal–sulfur bonds or π complexation interactions between metals and sulfur atoms in sulfur compounds, so that sulfur is attached to the adsorbent to achieve the goal of reducing sulfur content [105,106,107]. More importantly, metal oxides are highly efficient as most of the sulfides are easily adsorbed at Lewis active sites during the desulfurization process, reducing the mass fraction of sulfides in the fuel to less than 2.5 × 10^−5^ [108]. When metal oxides are used alone, the specific surface area is generally small and the utilization rate is not high, so they are generally loaded on the surface of inorganic oxides with larger specific surface areas to form supported metal oxides or composite metal oxides for desulfurization research. For example, Zheng et al. [109] synthesized Ag-CeOx/TiO_2_-SiO_2_ adsorption materials modified by B_2_O_3_. The adsorption results showed that the addition of B_2_O_3_ promoted the dispersion of CeOx species, and then promoted the dispersion and oxidation of Ag species, thus improving the adsorption desulfurization activity. Among them, Ag_2_O and Ag_2_O_2_ are the main active substances. Yin et al. [110] prepared a series of new aerogel composites of CeO_2_/SiO_2_ with different Si/Ce molar ratios by a simple and energy-saving sol-gel method, and investigated their adsorption desulfurization performance (see Figure 13). The experimental results show that the adsorption process of CeO_2_/SiO_2_ conforms to quasi-second-order kinetics, and the Langmuir and Freundlich models are well fitted. At the same time, there is a weak Lewis acid site on the CeO_2_/SiO_2_ surface, which is also the main factor in improving the adsorption performance. As the active component, metal oxides can combine well with organic sulfides. Compared with single metal oxide adsorbents, mixed metal oxides are also used in adsorption desulfurization because of their better metal synergies. In addition, they found that the adsorption capacity of the regenerated adsorbent for TP decreased, mainly due to the anchoring of partially added Ce to the adsorbent surface through the formation of Si-O-Ce bonds. The Ce content of CeO_2_/SiO_2_-25 regenerated after the fourth cycle was 81% of that of fresh CeO_2_/SiO_2_-25.

Metal oxide adsorbents are widely used in ADS because of their simple synthesis route, insolubility in common solvents, and good structural stability. As an active component, metal oxides can selectively bind to organic sulfides despite their small sulfur adsorption capacity. Therefore, they are used for the modification of other adsorbents. It is worth noting that, although metal oxides have good adsorption desulfurization performance, they strongly interact with organic sulfides, which lead to complex processes and a large energy consumption being involved in the regeneration process, resulting in high production costs.

### 3.5. Other Adsorption Materials

**Figure 14:**
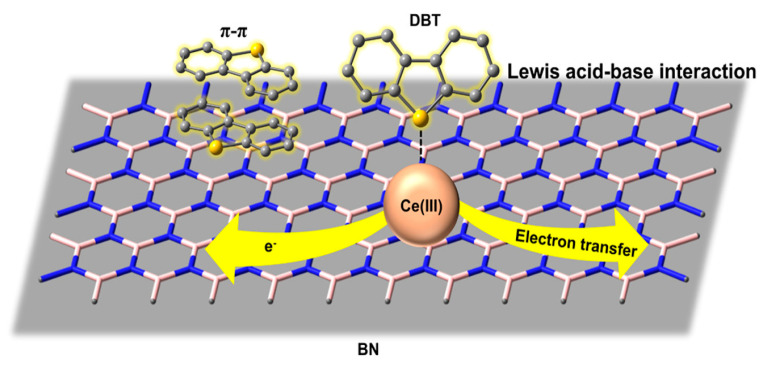
Possible adsorption mechanism schematic. Reprinted with permission from American Chemical Society [112].

In recent years, many two-dimensional (2D) materials, such as graphene, graphyne, MXene (X refers to group IV, V, or VI elements), and boron nitride, have attracted attention due to their unique structures and properties. Among them, boron nitride (BN) stands out due to its honeycomb atomic structure, oxidative stability, and strong regeneration capability. For instance, Yan et al. [111] found that porous BN microfibers have excellent adsorption desulfurization performance. The adsorption capacity of DBT could reach 86 mg S/g. Interestingly, the adsorbed porous BN could be regenerated by simple heat treatment. Inspired by their study, Luo et al. [112] developed a Lewis acidic BN for DBT (500 ppm S) desulfurization by adding Ce (III). The new Lewis acidic sites formed by two neighboring Ce (III) in this adsorbent enhanced the overall acidity. Meanwhile, utilizing the Lewis acid–base interaction between dibenzothiophene and the adsorbent acid sites, the adsorbent showed an excellent adsorption capacity with a sulfur capacity of up to 48.4 mg S/g. Furthermore, the strong Lewis acid sites in the adsorbent could also alleviate the competitive adsorption of aromatics and improve the selectivity of dibenzothiophene (see Figure 14). In recent years, Li et al. [113] developed a new CeVO4/BNNS adsorbent supported by boron nitride nanosheets. Under the conditions of reaction temperature of 130 °C, a catalyst dosage of 0.33 g, a simulated oil dosage of 110 mL, and an air pump flow rate of 150 mL/min, the removal rates of 4,6-DMDBT, DBT, and BT reached 100%, 99.62%, and 71.29%, respectively. At the same time, the adsorbent has good recycling and regeneration performances. The desulfurization rate of CeVO_4_/BNNS can still reach above 91% after being recycled six times.

**Figure 15:**
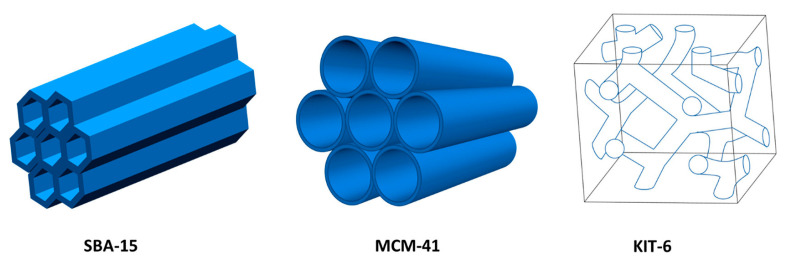
SBA-15, MCM-41, and KIT-6 mesoporous silica structures Reprinted with permission from American Chemical Society [2].

**Figure 16:**
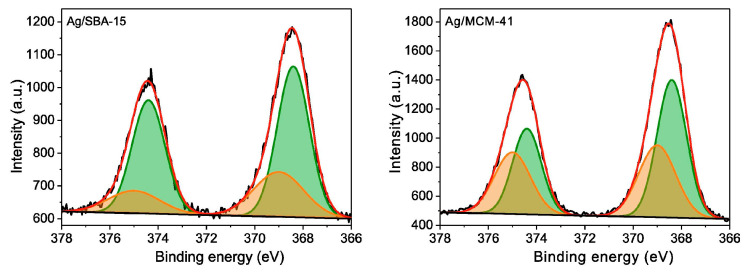
The XPS spectra of Ag 3d region for Ag/SBA-15 and Ag/MCM-41 sorbents along with the peak fitting of different forms of Ag. Reprinted with per-mission from Elsevier [117].

Mesoporous silica, as one of the most widely studied ordered mesoporous materials, has the advantages of an adjustable pore structure and a large specific surface area (700–1300 m^2^/g) [114,115,116]. Therefore, mesoporous silica has unique properties in adsorption desulfurization. Figure 15 illustrates several common types of mesoporous silica, including SBA-15, MCM-41, and KIT-6 [2]. However, the ion exchange capacity of mesoporous silica is limited due to a weak surface interaction. Accordingly, it is necessary to modify them. Recently, incorporating active metals into carriers has provided new ideas for the modification of mesoporous silica. Liu et al. [117] successfully obtained Ag/SBA-15 and Ag/MCM-41 adsorbents by using SBA-15 and MCM-41 mesoporous materials loaded with silver. The XPS results showed that the silver in Ag/SBA-15 and Ag/MCM-41 adsorbents were dominated by metallic silver, and some of the silver strongly interacted with the carriers to form Ag-O-Si bonds, which act as the main active center in the thiophene compounds. The binding energies of Ag 3d_5/2_ and 3d_3/2_ orbitals in Ag/SBA-15 and Ag/MCM-41, corresponding to Ag nanoparticles and metallic “bulk” Ag, were centered at 368.4 and 374.4 eV due to small charge effect. The other pairs of orbitals at higher binding energies of 369.0 and 375.0 eV were attributed to more oxidized surface Ag atoms being bonded to the surfaces of SBA-15 and MCM-41 (Ag-O-Si) due to a large charge effect (see Figure 16). The adsorption experiments demonstrated that the desulfurization efficiency of Ag/SBA-15 and Ag/MCM-41 adsorbents increased with the increase in Ag-O-Si content. Interestingly, due to the fact that TP can be efficiently adsorbed to the Brønsted acidic sites of SBA-15 and MCM-41 via S-H bonds, they exhibit a high level of selectivity in thiophene-cyclohexane and tetrahydrothiophene-cyclohexane solutions. A summary of various mesoporous silica adsorption materials performance for ADS was shown in Table 4.

At present, due to the unique physical structures and properties of mesoporous silica adsorption materials, it is easy to chemically react with the target substance or produce adhesion during the adsorption process, resulting in a limited regeneration of the adsorbent and a limited service life. The recycling and reuse of adsorbents still needs to be further explored.

Ionic liquids have a wide range of applications in green chemistry, such as liquid–liquid extraction, gas separation, electrochemistry, and catalysis, due to their properties of non-volatility, non-flammability, and high thermal stability [124,125,126]. In recent years, ionic liquids, as a new type of liquid adsorbent, have been gradually applied to the study of the adsorption desulfurization of fuel oil [127]. This is mainly because ionic liquids can be separated from sulfur-containing compounds by distillation or dissolution in water to achieve regeneration [128]. In this process, the ionic liquid remains stable and is not easily corroded. Therefore, ionic liquid can be used as an adsorbent in fuel adsorption desulfurization applications many times. Since ionic liquids are expensive, they are usually loaded onto MOFs, metal oxides, or carbon materials to realize their adsorption properties. For instance, Song et al. [129] successfully synthesized a highly active and stable MOF-199-loaded ionic liquid [Hnmp] [H_2_PO_4_] (IL-NP) adsorbent (Py/MOF). The adsorption experiments showed that the ADS activity of MOF-199 was improved after the introduction of the ionic liquids, which was mainly due to the improvement of the close contact between the sulfur compounds and the adsorbent as well as the increase in the adsorbed acidic sites for the weakly basic sulfur compounds. The effects of water, toluene, cyclohexene, and ethanol on the ADS activity of the synthesized adsorbent were also investigated. After adding MOF-199 and Py/MOF adsorbents to model gasoline, the ADS activity of the DBT molecules decreased. MOF199 is bound to sulfur compounds by π complexation and an acid–base coordination bond, and aromatics can also be adsorbed by π electron cloud interaction competition. However, all of these tested interfering compounds had relatively lower effects on desulphurization on Py/MOF adsorbents than MOF-199, possibly as a contribution to the additional acid sites introduced by acidic ionic liquids. Last, the removal of DBT on Py/MOF decreased by only 4.9% compared with that of fresh adsorbent after four cycles of regeneration. In another work, metal-based ionic liquids were introduced into inorganic materials as metal sources. Xiong et al. [130] prepared mesoporous Cu/SiO_2_ (Cu-IL) adsorbent materials with excellent adsorption and desulfurization performances by introducing Cu metal active sites into an Si matrix using the ionic liquid [C(16)mim]Cl/CuCl_2_ as a template. Since the metal active sites were introduced into the mesoporous matrix by the in situ-generated metal-based ionic liquid, they could be highly dispersed in the mesoporous skeleton without agglomeration.

As mentioned above, different adsorbents have different adsorption properties and selectivity. Generally, the adsorption properties of adsorbents depend on their own chemical composition, surface physical, and chemical properties, such as active sites and density, specific surface area, distribution of pore size, functional groups, and so on. The most fundamental influence is the surface physical and chemical properties of the adsorbent, which are crucial for the design and synthesis of highly selective adsorbents.

## 4. Regeneration Performance

The regeneration performance of an adsorbent directly affects the service life of the adsorbent and is an important factor in evaluating the performance and feasibility of the adsorption desulfurization process. Heat treatment of an Fe_3_O_4_@C adsorbent material was carried out by Tan et al. [131]. Adsorption studies have shown that the adsorbent can be easily separated from the adsorption system by an external magnetic field in a short period of time and exhibits excellent properties comparable to those of fresh adsorbents. More importantly, the use of magnetic core–shell structure adsorbents greatly simplifies the regeneration process and prevents mass loss during the recovery process. As mentioned previously, an excellent regeneration performance was also obtained for Meso-NiCe adsorbent materials under calcined conditions [71]. After the second cycle, the regeneration rates of thiophene and dibenzothiophene were 83.4% and 89.8%, respectively. Similarly, Ahmadi et al. [69] also regenerated the adsorbed CoO-MSU-S by calcination in air at 350 °C for 10 h, followed by activation/autoreduction in flowing helium at 450 °C for 18 h and then cooling the bed. The CoO-MSU-S regenerated by this method showed excellent activity in cycling experiments. Recently, Li et al. [107] studied the adsorption and regeneration properties of a NiSO_4_/ZnO-Al_2_O_3_-SiO_2_ mixed metal oxide adsorbent on dibenzothiophene in simulated oil with 500 ppm sulfur content and 328.3 ppm FCC gasoline. ZnO and Al_2_O_3_ could provide the Lewis acid site for the adsorbent to bind to the organic sulfide, while the NiSO_4_ component could ensure the stability of the adsorbent regeneration process. The used adsorbent was regenerated at 360 °C for 30 min and then at 480 °C for 1 h. After five cycles of desulphurization, the adsorbent maintained a high desulphurization activity on FCC gasoline. Surprisingly, the desulphurization activity of the adsorbent can be effectively recovered by corresponding regeneration operations. This mixed metal oxide adsorbent reduced the sulfur concentration of analog oil and FCC gasoline to 10 ppm.

## 5. Conclusions and Prospects

In conclusion, the sulfur in fuel oil is toxic, especially the sulfur dioxide produced by consuming it, which can form acid rain, damage the ozone layer, and cause problems such as the corrosion of refinery equipment. For human health and sustainable ecological development, the production of a low-sulfur “clean fuel” has become an inevitable trend. Adsorption desulfurization is considered to be a potential desulfurization technology. Adsorption materials are the core of ADS process, great advances have been made for several typical porous adsorption materials such as activated carbon materials, zeolite molecular sieves, metal-organic frameworks, and metal oxides for the ADS of liquid fuels. All of them have the advantages of a large specific surface area, an adjustable pore structure, and controllable surface properties. Among them, the molecular sieve is the most advantageous adsorbent material due to its excellent thermal stability, easy industrialization, and good regeneration performance. Although there are problems such as the adsorption capacity of sulfur and low selectivity, we believe that these problems will be gradually improved and solved in the near future. MOFs are one of the most promising adsorbent materials in recent years. However, high cost, low stability, and complex synthesis processes are the key points to be solved before industrialization. The development and application of new adsorbent materials for the deep desulfurization of fuel oil provide new options for reducing air pollution and protecting the environment.

However, the composition of the actual fuel oil is more complex, and the similarity of the molecular charge structures of olefin, aromatics and thiophene sulfide leads to the formation of a strong competitive adsorption on the active center of the adsorbent. Due to the complexity of adsorbent preparation and/or the price of the final adsorbent, and the possible difficulty of recycling, many adsorbents in the laboratory still have a long way to go before they can be used in industrial applications. Therefore, in the future, we can categorize different adsorption materials based on their sulfur molecule size or complexity, with the aim of designing high-stability, low-cost, and highly selective functional adsorbents, active adsorbents, and bifunctional active adsorbents. Additionally, reactors using multiple bed materials are also a good approach to consider.

Actually, the development of cost-effective adsorption desulfurization technology requires not only the innovation of reaction mechanism and materials, but also the improvement of desulfurization process. According to the current reports, combining adsorption desulfurization with oxidation desulfurization technology, thiophene sulfides are adsorbed first and then removed by oxidation to overcome some practical problems in adsorption desulfurization, which is also an efficient new desulfurization method. Finally, good reusability is also an advantage of ideal adsorbents. Therefore, it is necessary to explore the suitable regeneration methods for different adsorbents in the subsequent experimental process to improve the recycling performance of adsorbents. In the future, more attention should be paid to the regeneration and recycling of adsorbents to reduce the generation of waste and achieve the sustainable use of resources.

## Figures and Tables

**Table 1:** Overview of activated carbon materials for ADS.

Absorbents	Sulfur Source	Initial S Contents	Final S Contents	Performance	Ref.
AC/Mn/Cu	DBT	250–2500 ppm	16 ppm	136.78 mg/g	[55]
Mn/AC	DBT	200 ppm	<20 ppm	removal of 95.7%	[56]
AC	S-compounds	34.83	<10 ppm	----	[57]
3D-AC/Al_2_O_3_	feed oil	500 ppmw	<20 ppmw	96.6% sulfur removal	[58]
Ag/ZnONPS-AC	DBT	250 mg/L	----	removed 99%	[59]
AC-N-Mo	DBT	50–200 mg-S/L	-----	19.94 mg S/g	[60]

**Table 2:** Overview of molecular sieve materials for ADS.

Absorbents	Sulfur Source	Initial S Contents	Adsorption Capacity	Ref.
ZSM-5-based micro-/mesostructures	TP	50–450 ppm	14.1 mg/g	[72]
ZSM-5	TP	500 ppmw	0.0550 mmol S/g.	[68]
BL-ZSM-5	TP	100 ppmw	0.38 mg/g	[73]
ZSM-5	TP	500 ppm	0.48 mmol/g	[74]
Ag/TiO_2_-NaY (TY)	BT	10 mg/L	desulfurization rate of 95%	[75]
CuY1	synthetic gasoline	---	4.14 mg S/g	[76]
CuCeY	TP	500 mg/L	6.25 mg/g	[77]

**Table 3:** Adsorption capacity of MOFs reported in the literature.

Absorbents	Sulfur Source	Initial S Contents	Performance	Ref.
Ni-based MOF	TH	150 ppm	4.14 mg/g	[89]
Eu-MOF	TP/n-octane	1000 μg/g	24.59 mg S/g	[96]
MOF-199	TP	-----	72.575 mg/g	[97]
NHC/Co-MOF-Cu^2+^	DBT	100 mg/L	150.4 mg/g	[98]
MOF-5	DBT	1000 ppmw	31.9 mg S/g	[99]
HP-UiO-66-SO_3_Ag	BT	200–1000 ppm	31.4 mg S/g	[90]
MOF-5@AC	TP	100 μg/g	11.3 mg S/g	[100]
HPA-IL/ZIF-8	BT	1000 ppm	16.25 mg S/g	[101]

**Table 4:** Overview of mesoporous silica materials for ADS.

Absorbents	Sulfur Source	Initial S Contents	Final S Contents	Adsorbent Performance	Ref.
Ce-HY/SBA-15	TP	---	----	5.14 mg S/g	[118]
Ni_2_P/SBA-15	DBT	500 ppmw	----	11 mg S/g	[119]
Ag-Al/MCM-41	DBT	500 ppmw	<80 ppmw	84.6% DBT adsorbed fom model fuel	[120]
Co/MCM-41	actual diesel fuel	12,000 ppm	6700 ppm	reduced the sulfur content from 1.2 wt% to 0.67 wt%	[121]
Ce-KIT-6	TP	510 ppmw	<200 ppm	4.5 mg S/g	[122]
Ni-KIT-6	TP	517 ppmw	-----	6.25 mg S/g	[123]

## Data Availability

Not applicable.
